# Supplementary material for: The structure and function of P5A-ATPases
Source: Nat Commun. 2024 Nov 6;15:9605. doi: 10.1038/s41467-024-53757-6 (PMC11541931; doi:10.1038/s41467-024-53757-6)
Supplement: Supplementary file 1 — Supplementary Information [file 41467_2024_53757_MOESM1_ESM.pdf]

Supplementary Information for:

The structure and function of P5A-ATPases

Ping Li[1,†], Viktoria Bågenholm[2], Per Hägglund[2], Karin Lindkvist-Petersson[1], Kaituo Wang[2,3] & Pontus Gourdon[1,2,†]

[1] Department of Experimental Medical Science, Lund University, Sölvegatan 19, SE-221 84 Lund, Sweden

[2] Department of Biomedical Sciences, University of Copenhagen, Nørre Allé 14, DK-2200 Copenhagen N, Denmark

[3] Current address: State Key Laboratory of Plant Diversity and Specialty Crops, Institute of Botany, Chinese Academy of Sciences, Beijing, China

[†] Correspondence should be addressed to: P.L. (ping.li@med.lu.se) or P.G. (pontus.gourdon@med.lu.se)

Supplementary Fig. 1

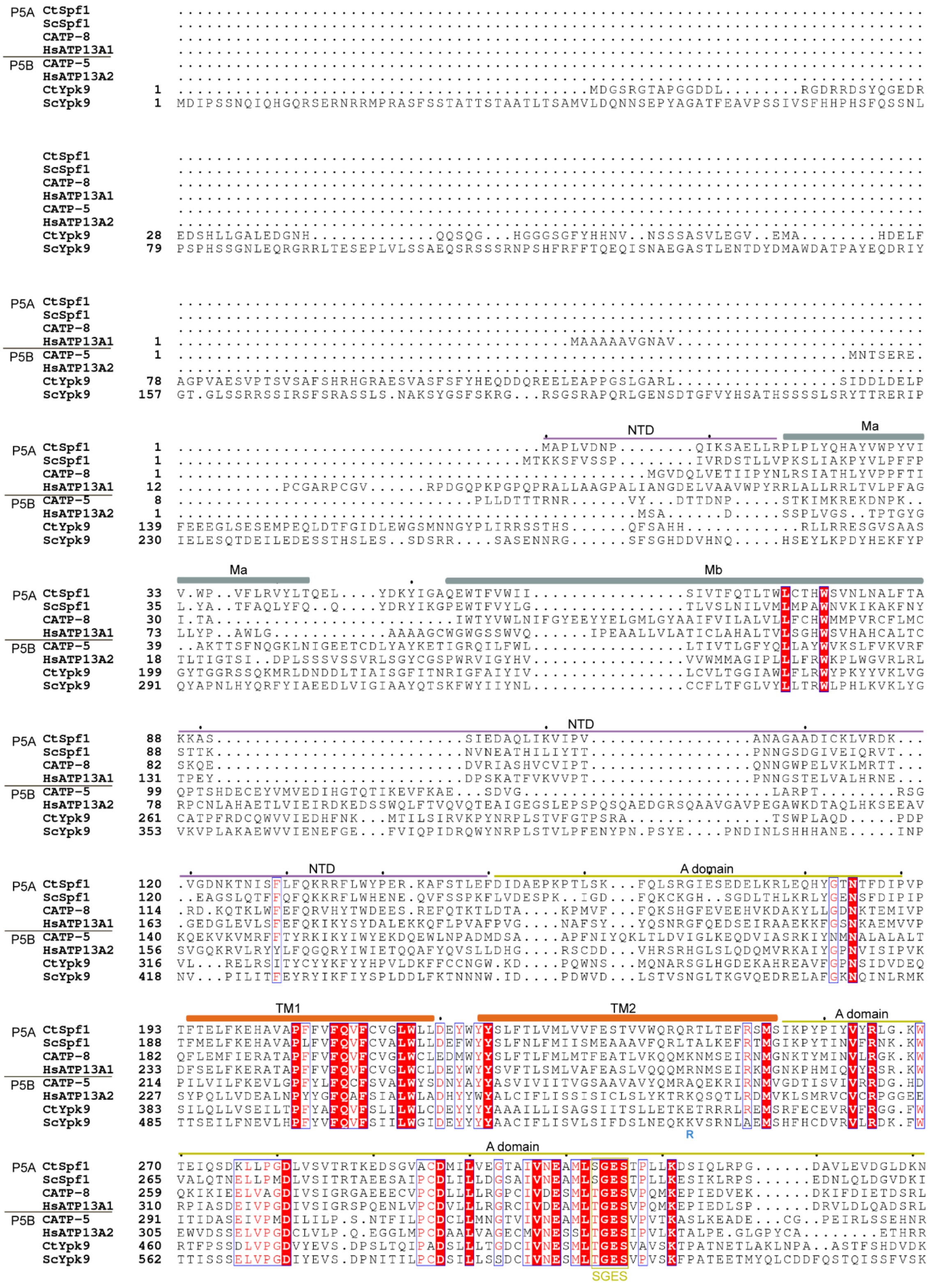

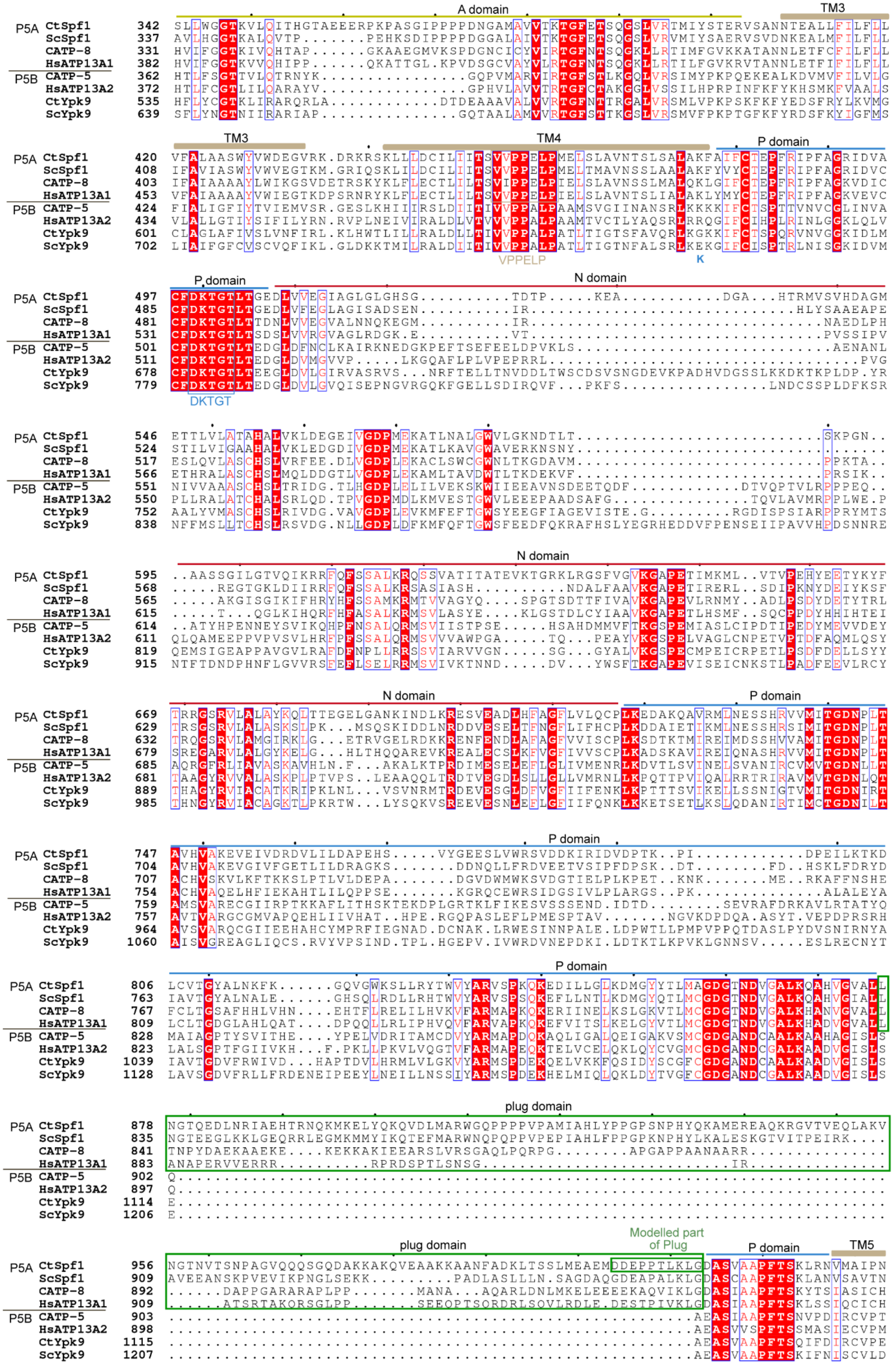
A domain
TM3
TM4
P domain
N domain
plug domain
Modelled part of Plug
TM5
VPPELP
K
DKTGT
P5A
P5B
CtSpf1
ScSpf1
CATP-8
HsATP13A1
CATP-5
HsATP13A2
CtYpk9
ScYpk9

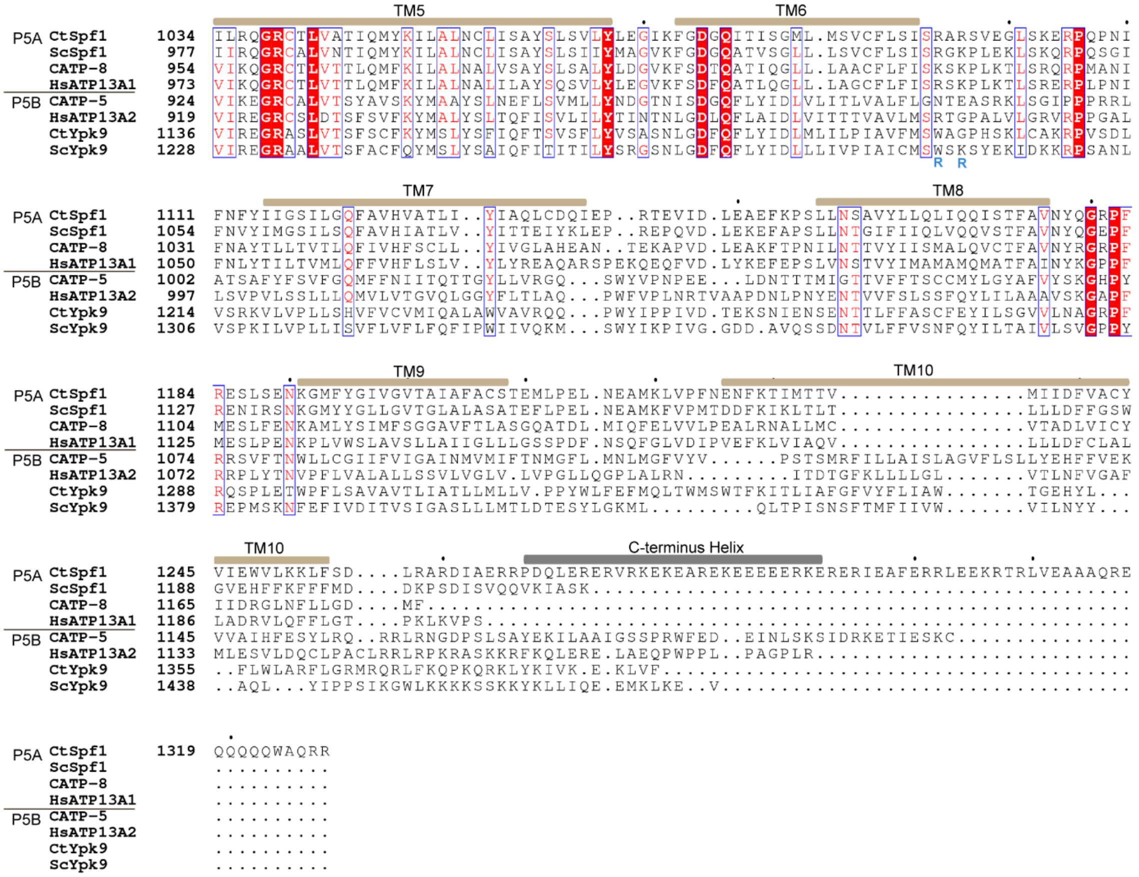

Supplementary Fig. 1. Structure-based multiple sequence alignment of selected P5A and P5B ATPases. The following sequences are included in the alignment (Uniprot ID in brackets) P5A: CtSpf1 (G0S4Z4), ScSpf1 (P39986), CATP-8 (P90747), HsATP13A1 (Q9HD20); P5B: CATP-5 (Q21286), HsATP13A2 (Q9NQ11), CtYpk9 (G0S7G9), ScYpk9 (Q12697). The alignment is annotated as follows: Purple line, NTD-domain (the soluble part). Yellow line, A-domain. Blue line, P-domain. Red line, N-domain. Gray cylinders, helices Ma and Mb. Orange cylinders, M1 and M2. Wheat cylinders, M3 to M10. Dark gray cylinder, C-terminal helix. Yellow box, conserved dephosphorylation loop (SGE). Blue box, conserved phosphorylation motif (DKTGT). Green box, Plug-domain. Wheat box, P5A conserved motif (PPELPM/IE) of M4. Positively charged residues (circled in blue), may contribute to cargo transport. Sequence alignments were performed using Cluster Omega (online) and visualized using ESPript 3.0 (online).

Supplementary Fig. 2

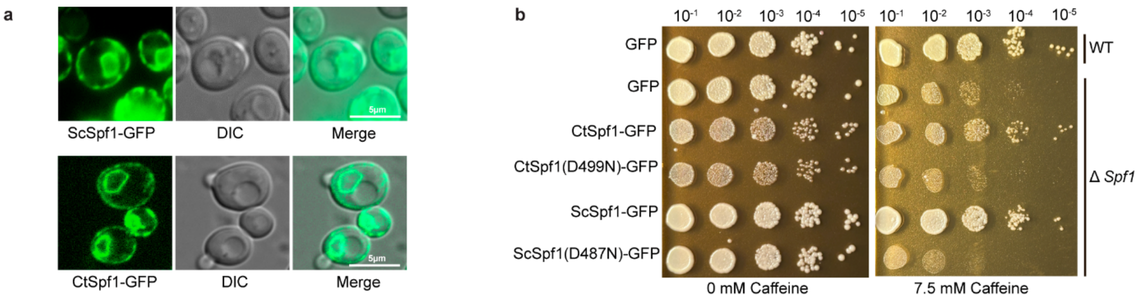

Supplementary Fig. 2. Functional characterization of CtSpf1. a Localization of CtSpf1 was investigated using fluorescence microscopy, comparing GFP-fluorescence (left), differential interference contrast (DIC) (middle) and merged (right), the scale bar represents 5 µm. Imaging experiments were conducted for three separate clones of each yeast strain. **b** Comparison of the growth on YPdG-agar plates of *S. cerevisiae* cells with wild-type CtSpf1-GFP, inactive CtSpf1-GFP (the D499N mutant), wild-type ScSpf1-GFP and inactive ScSpf1-GFP (the D487N mutant equivalent to the D499N mutant in CtSpf1) in the absence (left) or presence of caffeine (7.5 mM, right). GFP only constructs represent controls using the same plasmid as used for the P5A-ATPase forms. The experiment were performed at 30 °C in wild-type (WT, first row) and ScSpf1-deleted (all rows except for the first) BY4741 yeast strains. The growth assay was performed three times independently with similar results.

Supplementary Fig. 3

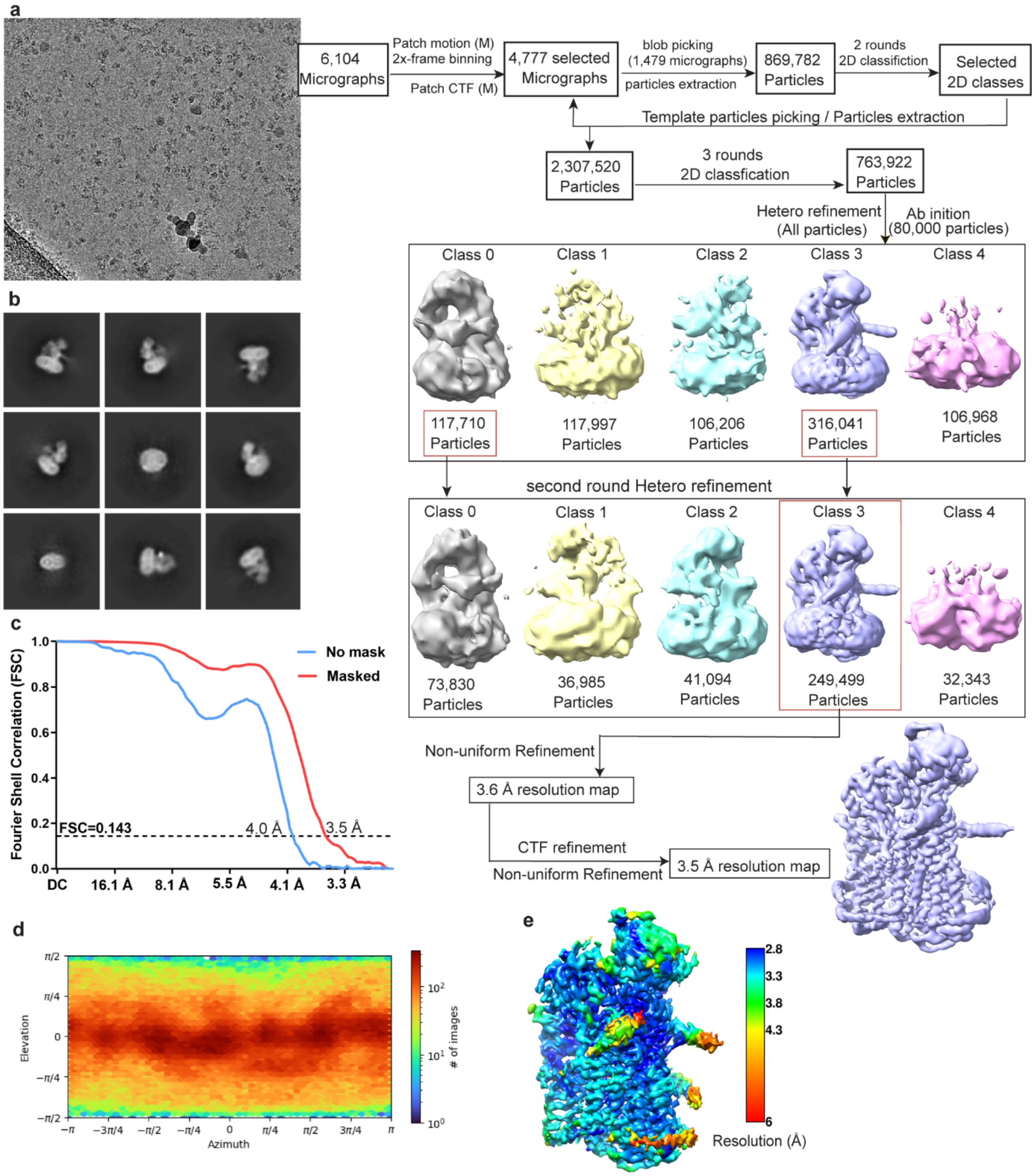

Supplementary Fig. 3. Cryo-EM data processing for the E2P state stabilized using BeF_3^-. **a** Representative motion corrected micrographs (4,777 micrographs were selected for further processing) and processing workflow. **b** Selected 2D classes with different views. **c** Gold standard Fourier shell correlation curves calculated for the reconstructed map based on a FSC 0.143 cut-off. **d** Particle orientation distributions of the final reconstruction. **e** Local resolution map based on a FSC 0.143 cut-off.

Supplementary Fig. 4

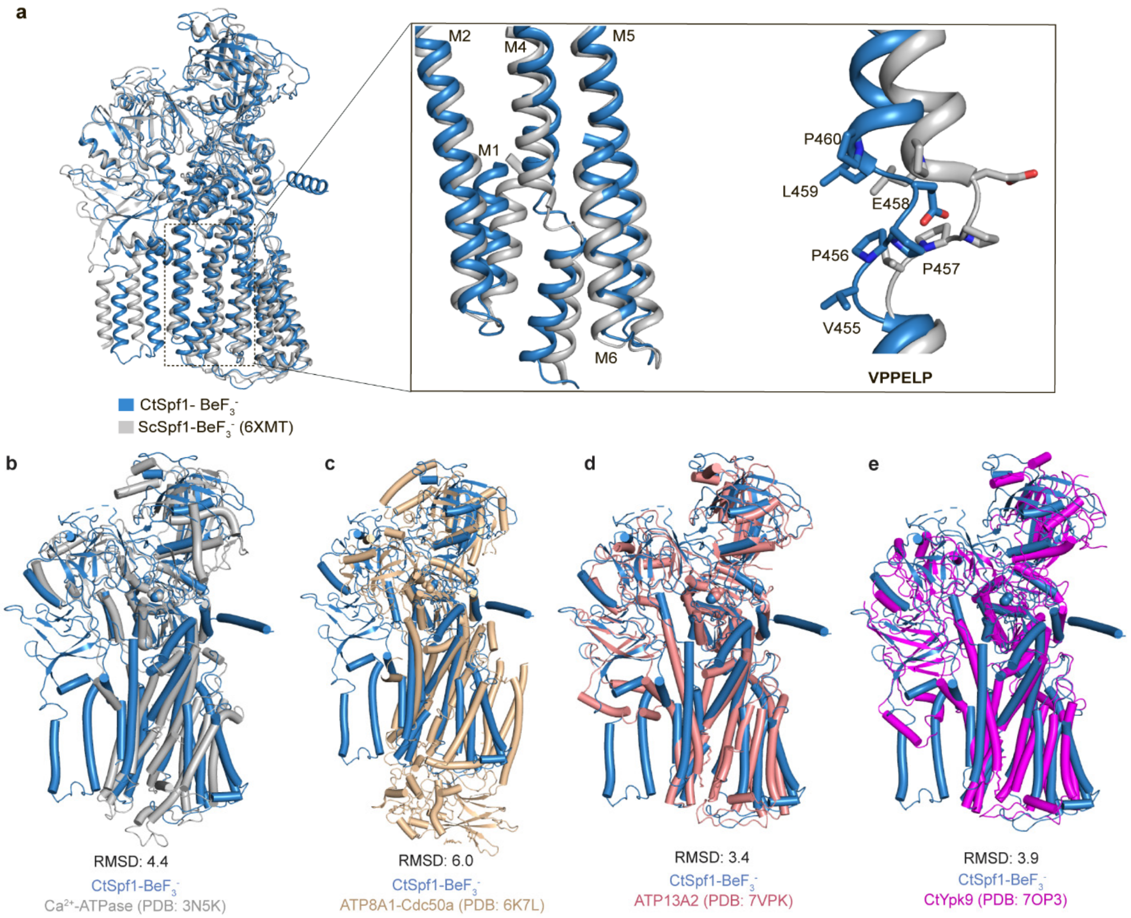

Supplementary Fig. 4. Structural comparisons of the E2P (BeF_3^--stabilized) state of the P5A-ATPase CtSpf1 with the corresponding conformations of ScSpf1 (another P5A-ATPase), a P2-ATPase, a P4-ATPase, and a P5B-ATPase. a Alignment of CtSpf1 (blue) with ScSpf1 (grey, PDB-ID 6XMT) with an overall RMSD of 2.8 Å. The different arrangement of the invariant P5A-motif PPELPM/IE of M4, which adopts a more peripheral location in ScSpf1 is shown in the close-view to the right. **b** Alignment of CtSpf1 (blue) with the Ca^{2+}-ATPase SERCA (grey, PDB-ID 3N5K) with overall RMSD 4.4 Å. **c** Alignment of CtSpf1 (blue) with ATP8A1-Cdc50a (wheat, PDB-ID 6K7L) with overall RMSD 6.0 Å. **d** Alignment of CtSpf1 (blue) with ATP13A2 (salmon, PDB-ID 7VPK) with overall RMSD 3.4 Å. **e** Alignment of CtSpf1 (blue) with CtYpk9 (pink, PDB-ID 7OP3) with overall RMSD 3.9 Å.

Supplementary Fig. 5

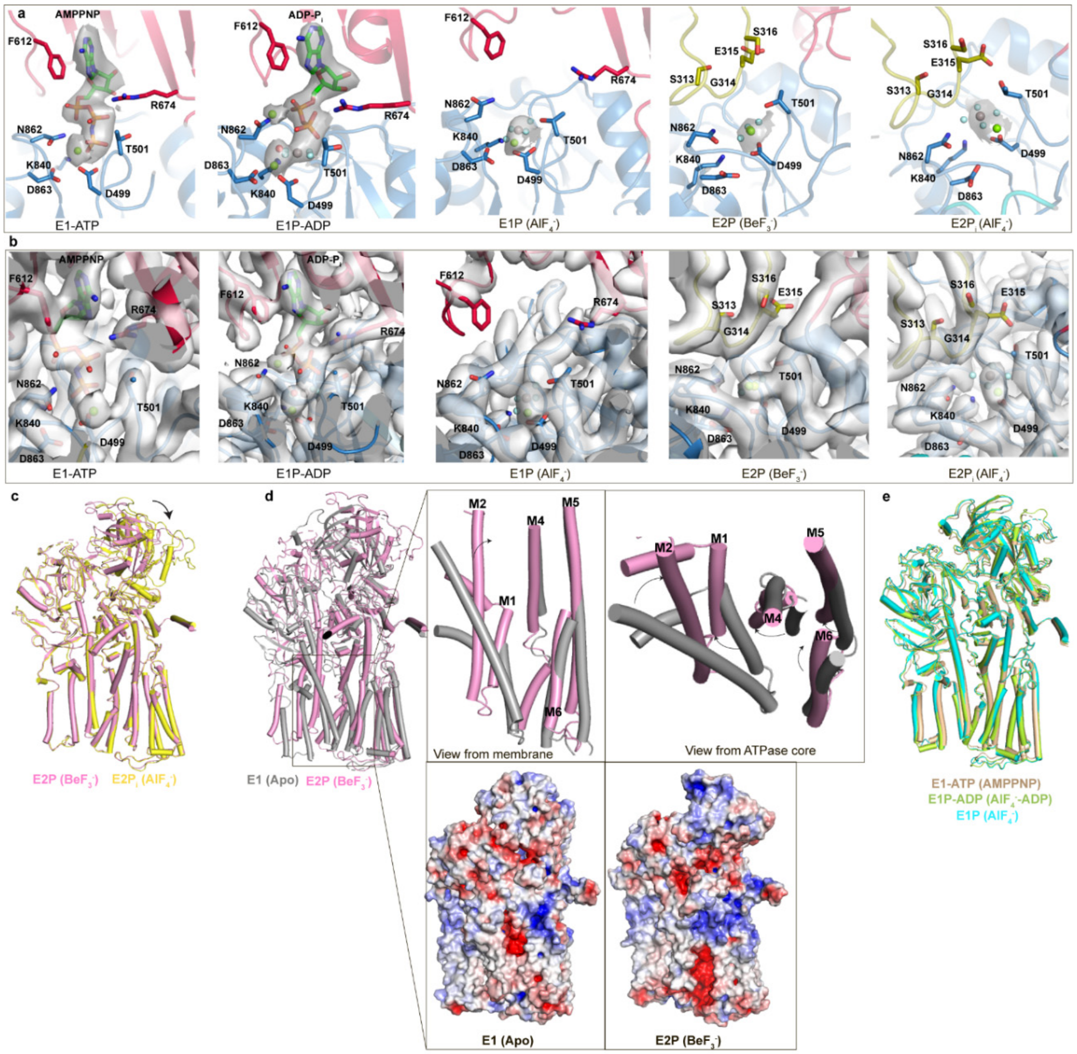

Supplementary Fig. 5. Structural comparisons of different states of CtSpf1. a The phosphorylation sites in each state. AMPPNP and ADP are shown as sticks. AlF_4^- (grey) and BeF_3^- (grey), Mg^{2+} (green) and H_2O (red) are depicted as spheres. Non-protein cryo-EM density is shown in grey. **b** Map and model of the phosphorylation sites in each state as in panel a but with cryo-EM density also for the surrounding region. **c** Structural comparison of the E2P and $E2.P_i$ states, revealing movement of the N-domain while the membrane spanning M-domains are more similar. **d** Structural comparison of the E1 (apo) and E2P states. The close-views display the movement of the transmembrane segments, as aligned on the P-domain and M5-M10. The transport pathway, that is partially lined by M1-M6, deviates substantially between the E1 (with a cytosol-facing cavity) and E2P (the all-through the membrane cleft) state. **e** Comparison of the E1P, E1P-ADP and E1P states.

Supplementary Fig. 6

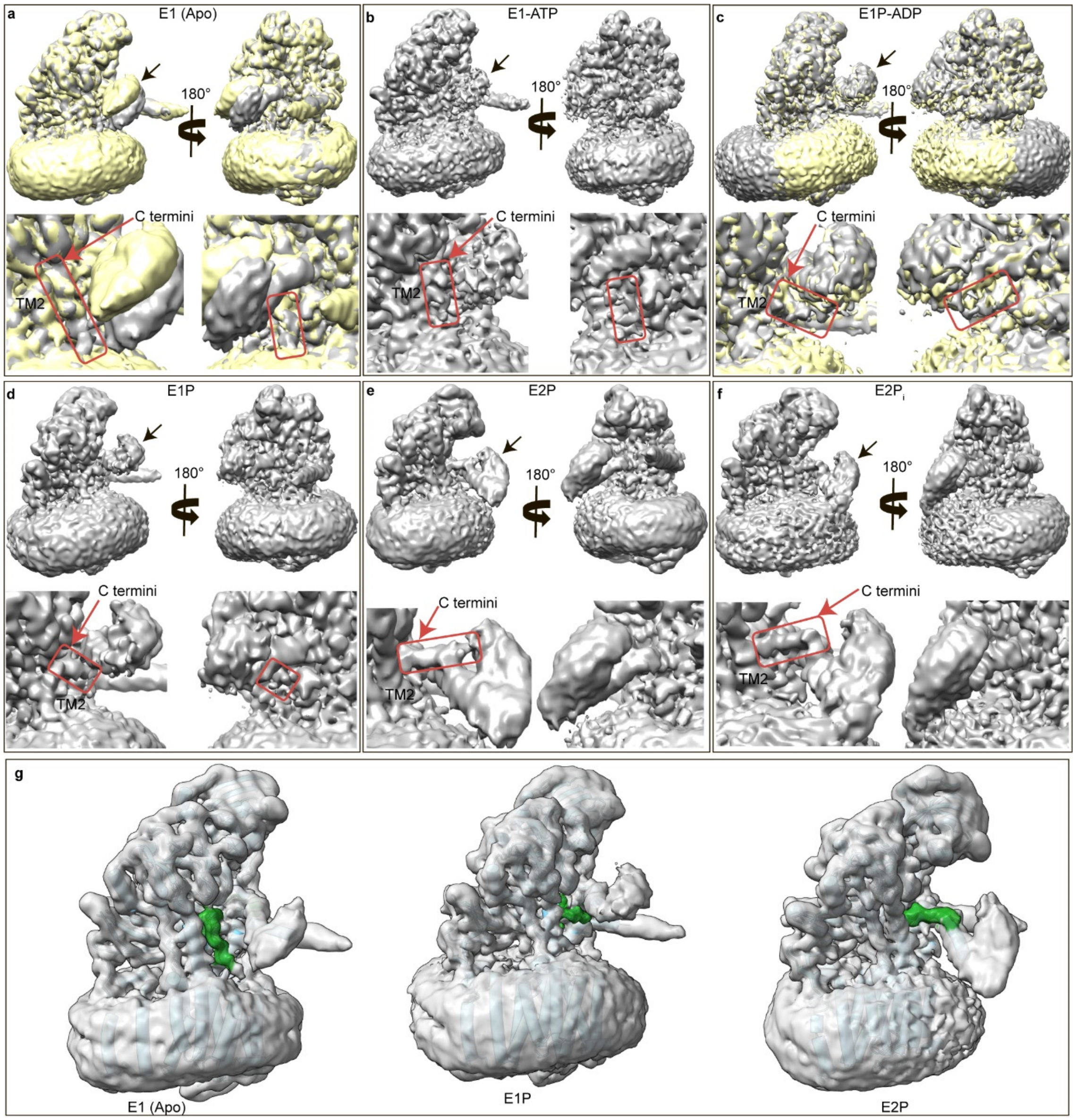

Supplementary Fig. 6. The P-domain insertion (Plug-domain) in the different structurally determined states. The Plug-domain is highlighted with black arrows. The C-terminal part of the Plug-domain is marked with a red box, as also pinpointed with red arrows. **a** The E1 (apo) state with two reconstructed maps, determined at 3.4 Å (grey) and 3.5 Å (yellow), see Supplementary Fig. 7. **b** The E1-ATP state. **c** Maps of the E1P-ADP state with the feature in the membrane (grey) and without membrane-feature map (yellow), see Supplementary Fig. 11. **d** The E1P state. **e** The E2P state. **f** The E2.P$_i$ state. **g** Modelled CtSpf1 structures (E1, E1P and E2P in cyan) with non-sharpened cryo-EM maps (grey and green) and with the C-termini of the Plug-domain is highlighted in green.

Supplementary Fig. 7

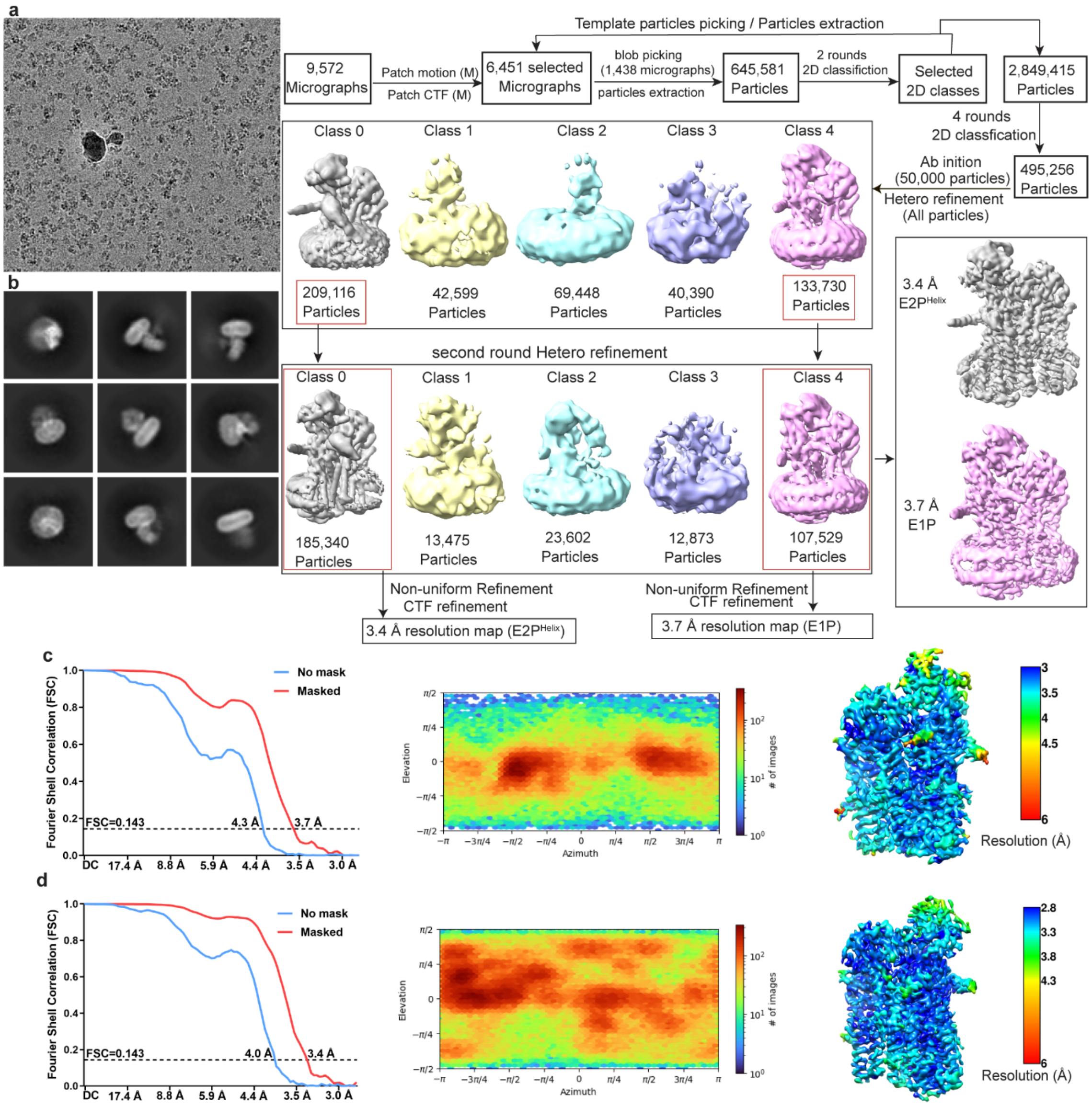

Supplementary Fig. 7. Cryo-EM data processing for the E1P and E2.P$_i$ states stabilized using AlF$_4^-$. a Representative motion corrected micrographs (6,451 micrographs were selected for further processing) and processing workflow. **b** Selected 2D classes with different views. **c** E1P: Gold standard Fourier shell correlation curves calculated for the reconstructed map based on a FSC 0.143 cut-off (left), particle orientation distribution of the final reconstruction (middle), and local resolution map (FSC 0.143) of the reconstructed map (right). **d** E2.P$_i^{cargo}$: Gold standard Fourier shell correlation curves calculated for the reconstructed map based on a FSC 0.143 cut-off (left), particle orientation distribution of the final reconstruction (middle), and local resolution map (FSC 0.143) of the reconstructed map (right).

Supplementary Fig. 8

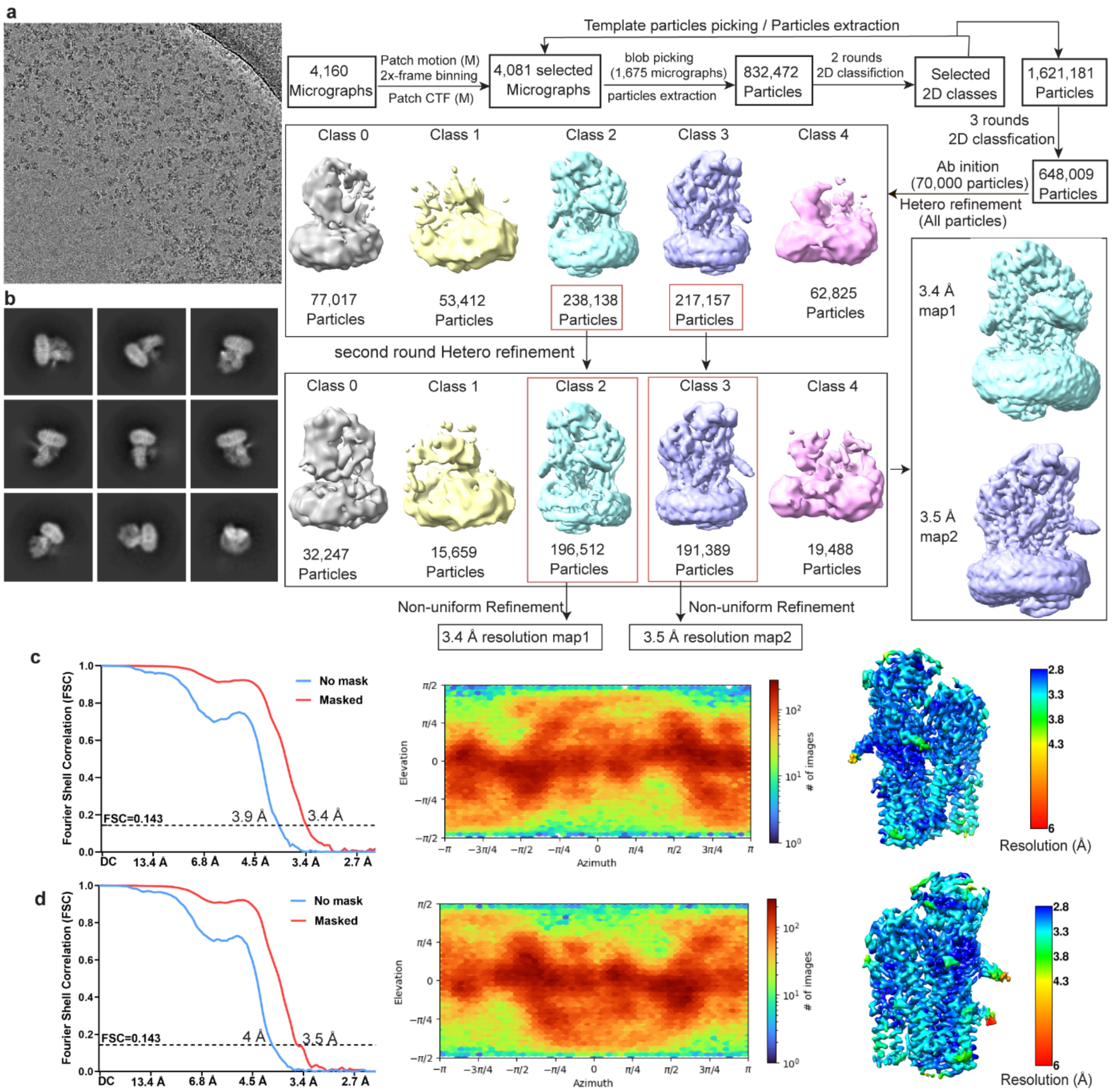

Supplementary Fig. 8. Cryo-EM data processing for E1 (apo). a Representative motion corrected micrographs (4,081) micrographs were selected for further processing) and processing workflow. **b** Selected 2D classes with different views. **c** map1: Gold standard Fourier shell correlation curves calculated for the reconstructed map based on an FSC 0.143 cut-off (left), particle orientation distribution of the final reconstruction (middle), and local resolution map (FSC 0.143) for the reconstructed map (right). **d** map2: Gold standard Fourier shell correlation curves calculated for the reconstructed map based on an FSC 0.143 cut-off (left), particle orientation distribution of the final reconstruction (middle), and local resolution map (FSC 0.143) for the reconstructed map (right).

Supplementary Fig. 9

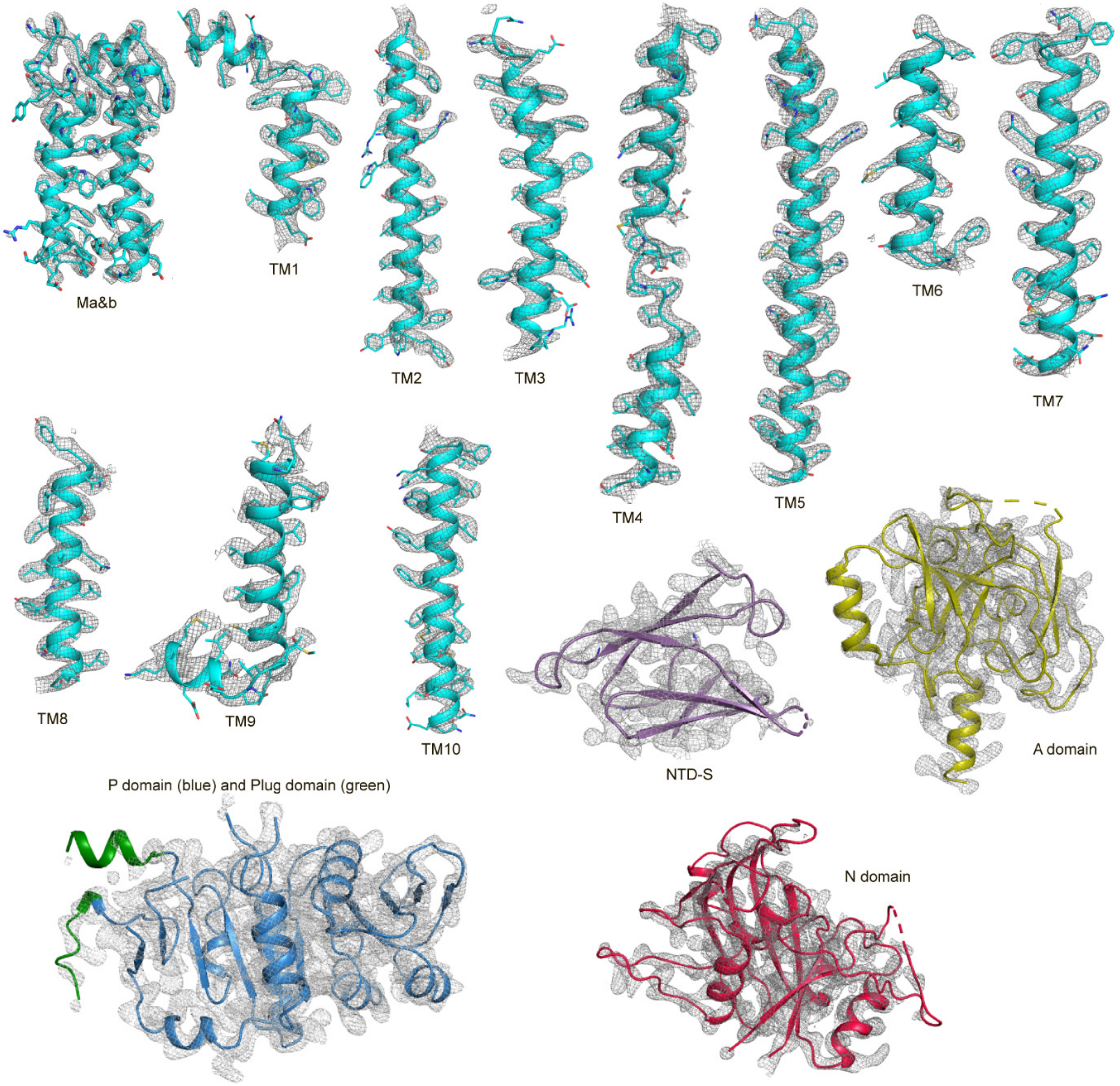

Supplementary Fig. 9. Cryo-EM density and associated model of representative parts of the E1 (apo) state. The transmembrane helices are shown in cartoon with stick sidechains. Soluble domains are shown in cartoon and the densities are show as grey mesh.

Supplementary Fig. 10

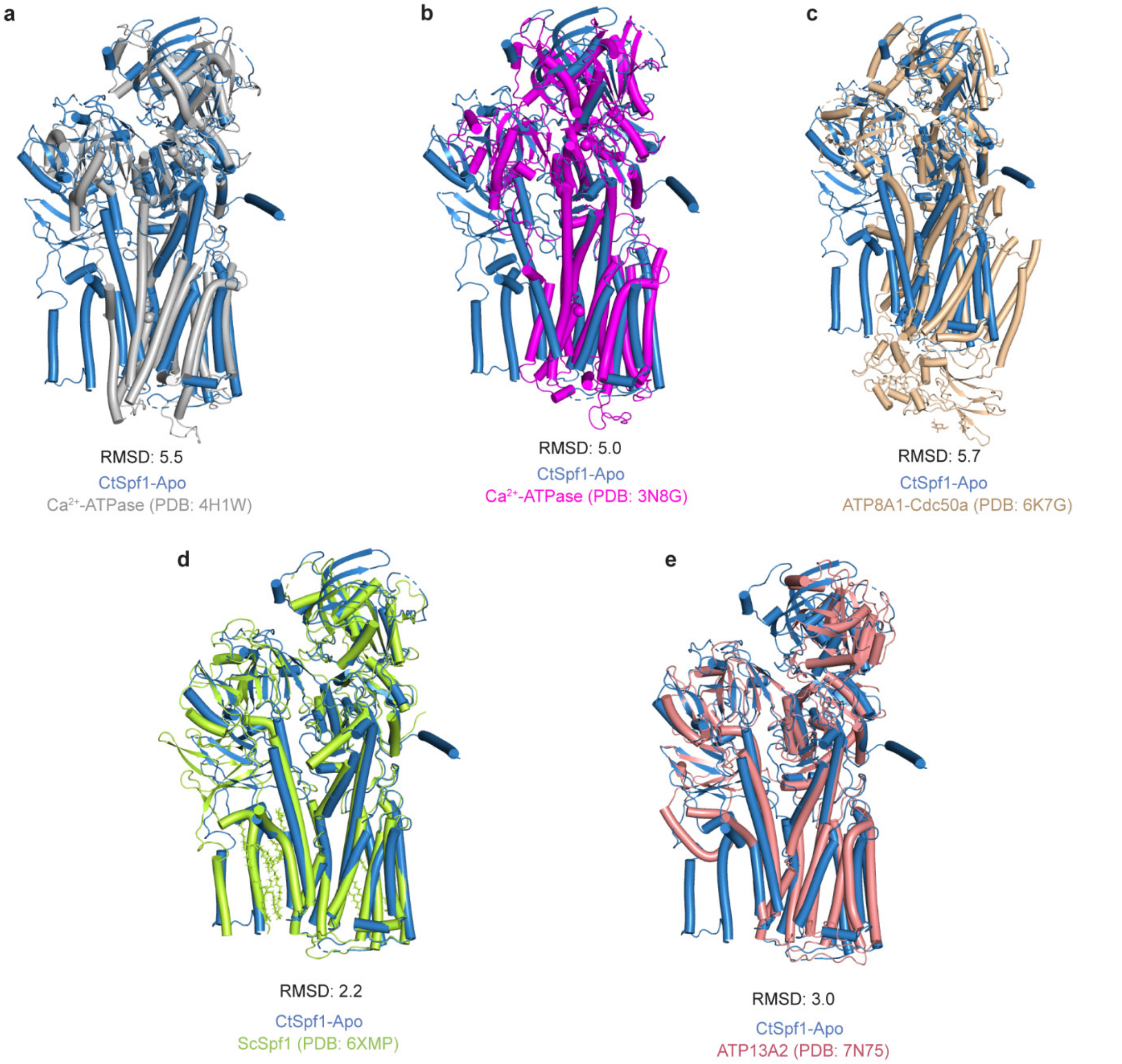

Supplementary Fig. 10. Structural comparisons of the E1 (apo) state of the P5A-ATPase CtSpf1 with relevant conformations of a P2-ATPase, a P4-ATPase, another P5A-ATPase (ScSpf1) and a P5B-ATPase. a Alignment of CtSpf1 (blue) with the Ca^{2+}-ATPase SERCA (grey, PDB-ID 4H1W). **b** Alignment of CtSpf1 (blue) with Ca^{2+}-ATPase (magenta, PDB-ID 3N8G). **c** Alignment of CtSpf1 (blue) with ATP8A1-Cdc50a (wheat, PDB-ID 6K7G). **d** Alignment of CtSpf1 (blue) with ScSpf1-Apo (limon, PDB-ID 6XMP). **e** Alignment of CtSpf1 (blue) with ATP13A2 (salmon, PDB-ID 7N75).

Supplementary Fig. 11

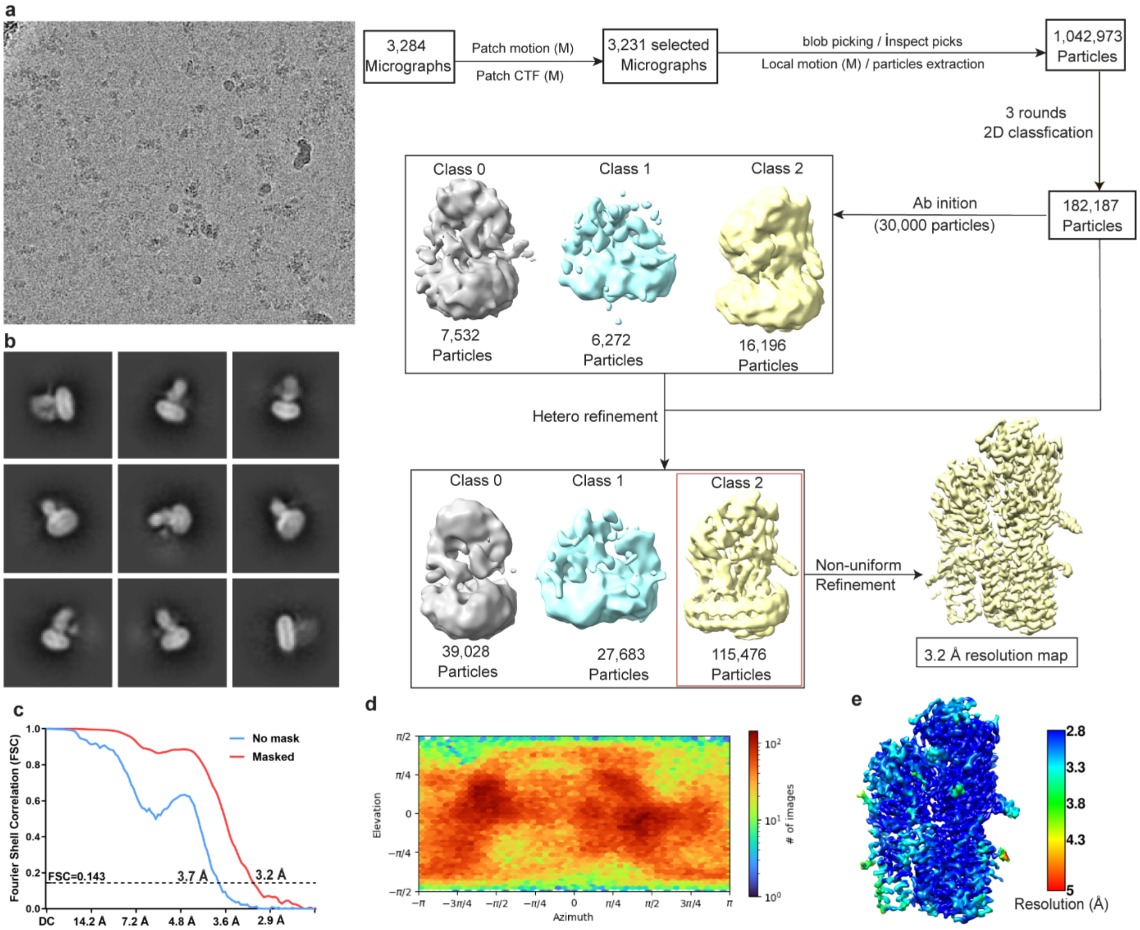

Supplementary Fig. 11. Cryo-EM data processing for the E1-ATP state stabilized by AMP-PNP. a Representative motion corrected micrographs (3,231 micrographs were selected for further processing) and processing workflow. **b** Selected 2D classes with different views. **c** Gold standard Fourier shell correlation curve calculated for the reconstructed map based on a FSC 0.143 cut-off. **d** Particle orientation distribution of the final reconstruction. **e** Local resolution map based on a FSC 0.143 cut-off.

Supplementary Fig. 12

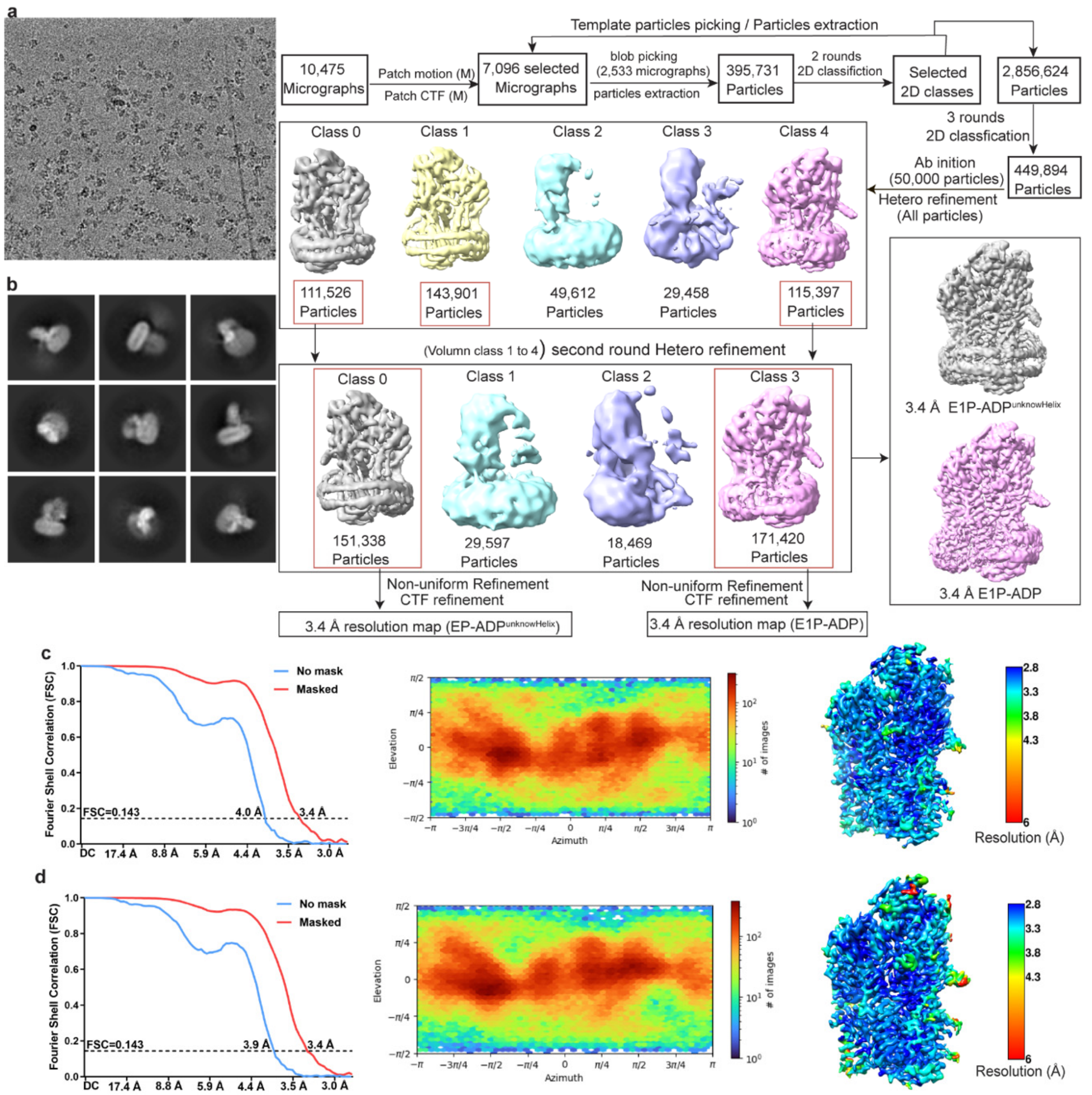

Supplementary Fig. 12. Cryo-EM data processing for the E1P-ADP and E1P-ADP$^{\text{membranous-feature}}$ states stabilized by AlF_4^- and ADP. a Representative motion corrected micrographs (7,096 micrographs were selected for further processing) and processing workflow. **b** Selected 2D classes with different views. **c** E1P-ADP$^{\text{membranous-feature}}$: Gold standard Fourier shell correlation curve calculated for the reconstructed map based on an FSC 0.143 cut-off (left), particle orientation distributions of the final reconstruction (middle) and local resolution map for the reconstructed map (FSC 0.143) with additional unassigned cryo-EM density (right). **d** E1P-ADP: Gold standard Fourier shell correlation curve calculated for the reconstructed map based on an FSC 0.143 cut-off (left), particle orientation distributions of the final reconstruction (middle) and local resolution map for the reconstructed map (FSC 0.143) without additional unassigned cryo-EM density (right).

Supplementary Fig. 13

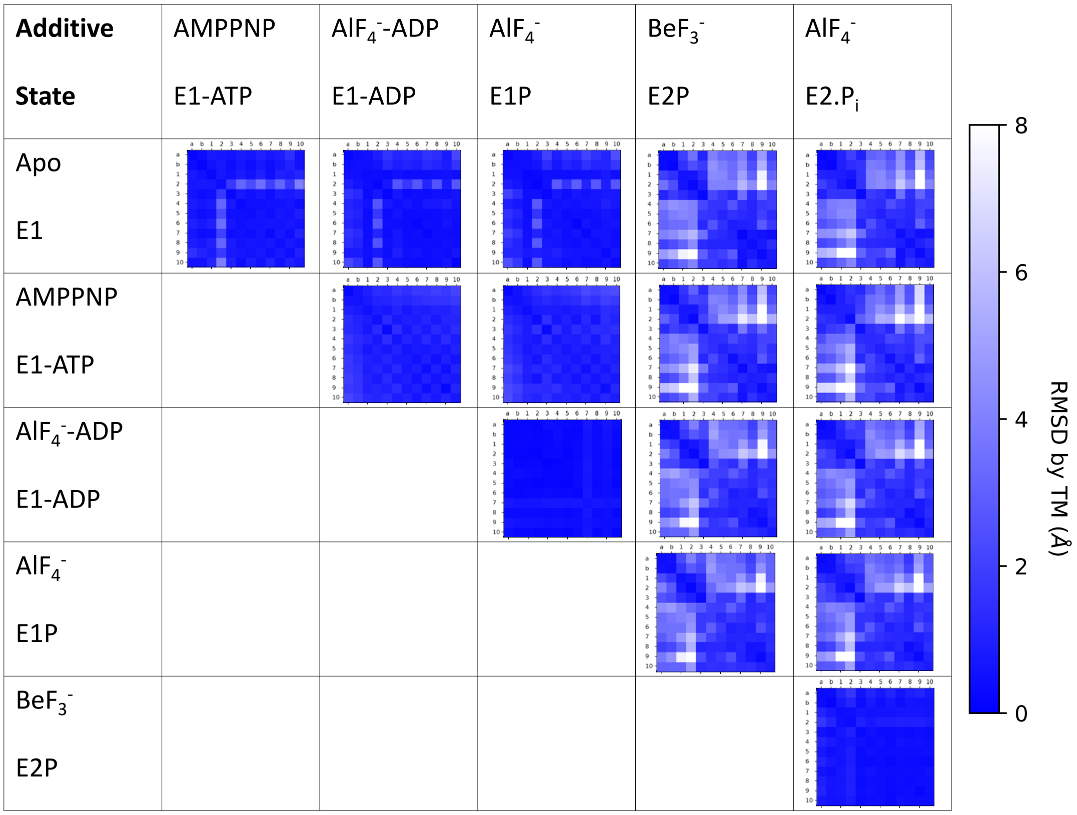

Supplementary Fig. 13. Distance difference matrices comparing the positions of TM helices across the different determined CtSpf1 structures. The analysis was conducted as previously reported, using overall alignments [1-3]. Each individual matrix shows relative differences in position of helices Ma, Mb, M1, M2, M3, etcetera from left to right and top to bottom. When comparing E1 states with E2P states the helices move as two blocks: Ma-M2 and M4-M10, with M3 remaining relatively stationary.

Supplementary Fig. 14

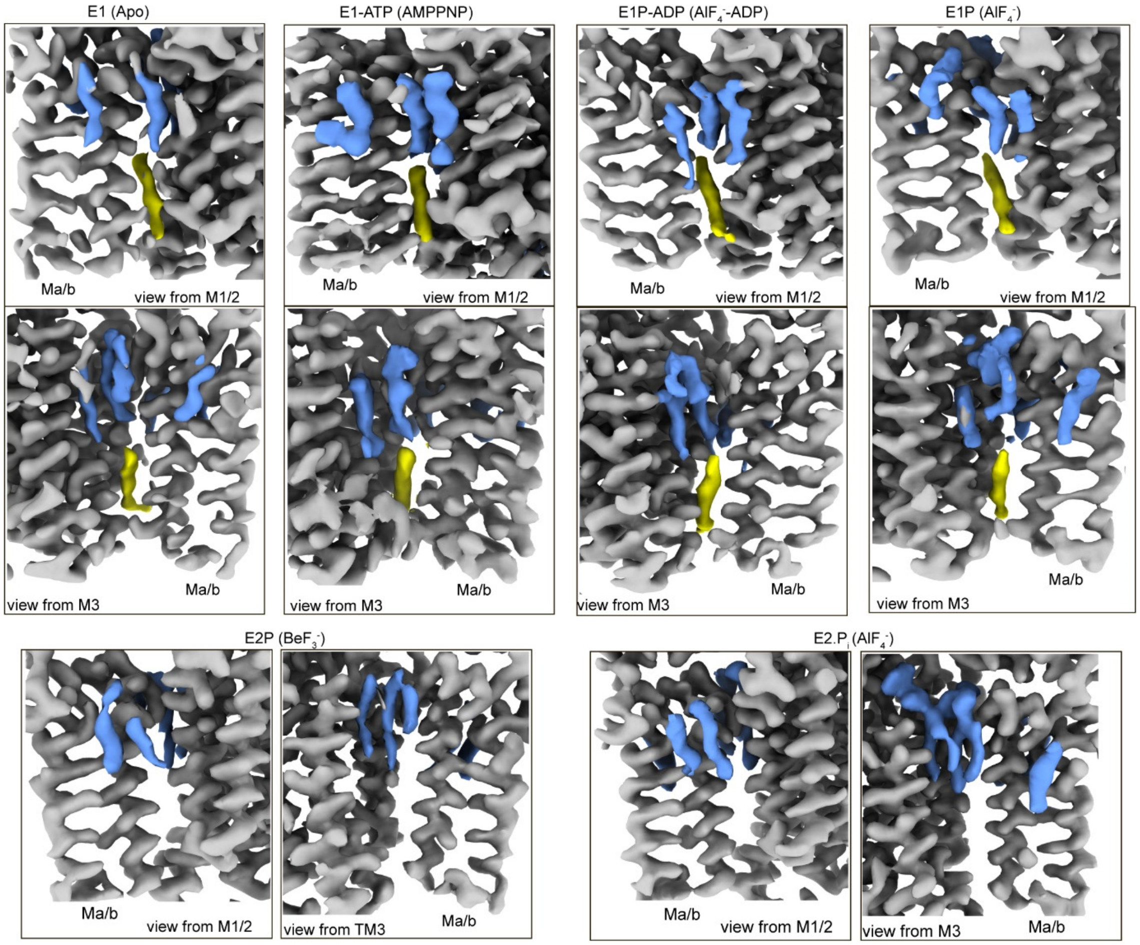

Supplementary Fig. 14. Identified cryo-EM densities that may represent lipids. Close-views of complementary non ATPase cryo-EM features. The blue densities close to the cytosol are present in all states. The yellow features are present in all E1, but not in the E2 states recovered here.

Supplementary Fig. 15

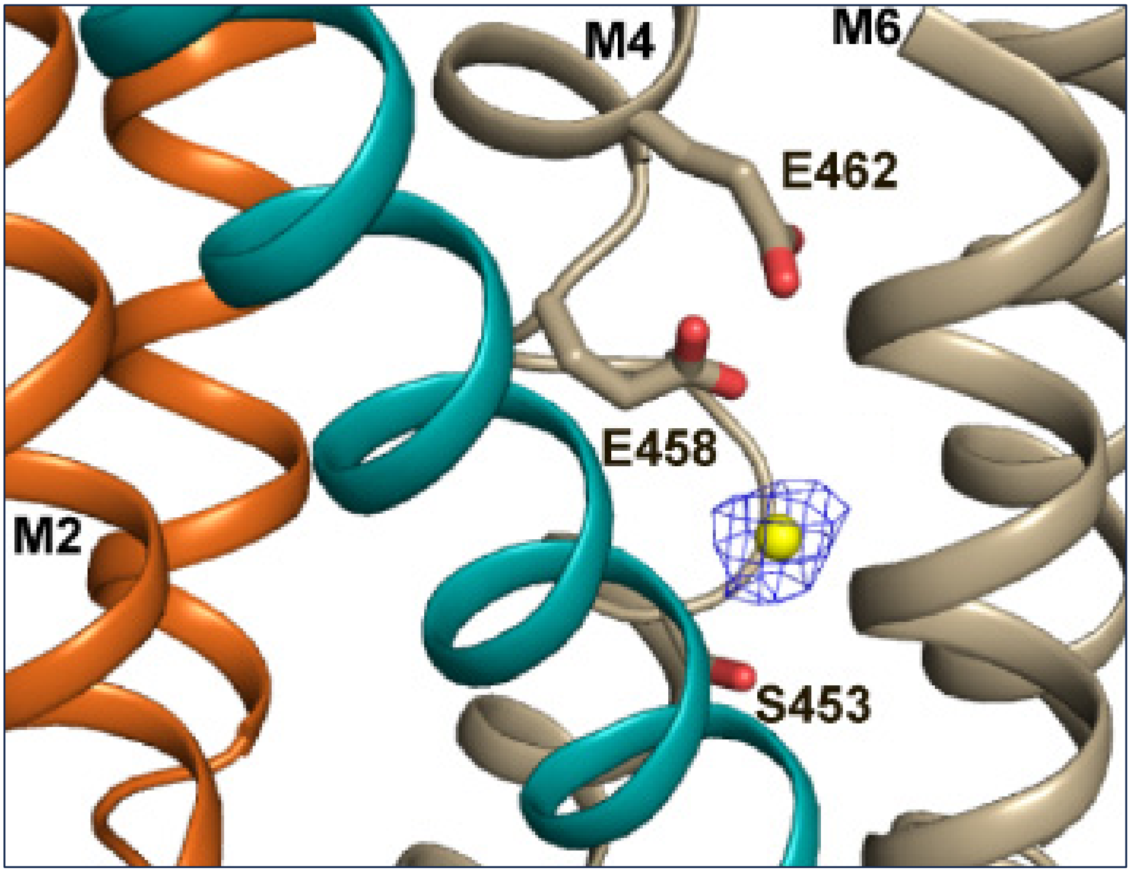

Supplementary Fig. 15. Unexplained cryo-EM density (blue) in E2.P_i structure.

Supplementary Table 1

**Spf1**	PDB ID	**6XMP**	**6XMQ**	**6XMS**	**6XMT**	**6XMU**
		Spf1 Apo	Spf1 (AMP-PCP)	Spf1 (AlF_4^-)	Spf1 (BeF_3^-)	Spf1 (BeF_3^-)<br>Endogenous substrate bound
Apo	**6XMP**	0.0	0.7	0.5	6.6	6.7
AMP-PCP bound	**6XMQ**	0.7	0.0	0.6	6.7	6.8
AlF_4^- bound	**6XMS**	0.5	0.6	0.0	6.5	6.6
BeF_3^- bound	**6XMT**	6.6	6.7	6.5	0.0	0.4
BeF_3^- and<br>Endogenous substrate bound	**6XMU**	6.7	6.8	6.6	0.4	0.0
**CtSpf1**						
Apo	**8OP3**	1.8	1.9	1.8	7.0	7.1
E1-ATP	**8OP4**	2.1	2.0	1.7	6.8	6.9
E1P:ADP	**8OP5**	1.6	1.4	1.2	6.6	6.7
E1P (AlF_4^-)	**8OP6**	1.7	1.4	1.4	6.4	6.5
E2-P (BeF_3^-)	**8OP7**	6.5	6.7	6.6	2.8	2.7
E2:Pi (AlF_4^-)	**8OP8**	6.2	6.4	6.3	4.2	4.2

**CtSpf1**	PDB ID	**8OP3**	**8OP4**	**8OP5**	**8OP6**	**8OP7**	**8OP8**
		Apo	E1-ATP	E1P:ADP	E1P (AlF_4^-)	E2-P (BeF_3^-)	E2:Pi (AlF_4^-)
Apo	**8OP3**	0.0	2.5	2.0	1.6	6.5	6.5
E1-ATP	**8OP4**	2.5	0.0	1.3	1.6	6.5	6.6
E1P:ADP	**8OP5**	2.0	1.3	0.0	0.6	6.7	6.7
E1P (AlF_4^-)	**8OP6**	1.6	1.6	0.6	0.0	6.6	6.7
E2-P (BeF_3^-)	**8OP7**	6.5	6.5	6.7	6.6	0.0	0.7
E2:Pi (AlF_4^-)	**8OP8**	6.5	6.6	6.7	6.7	0.7	0.0

Supplementary Table 1. Overall structural alignments of previously available (of CsSpf1) and here determined (CtSpf1) structures of P5A-ATPases. RMSD (Å) values are indicated. Homologous structures (RMSD < 1 Å) are highlighted in grey and blue, respectively. The most similar structures between CsSpf1 and CtSpf1 are indicated in green.

Supplementary Table 2

	E1 (Apo) (EMDB-17039) (PDB-8OP3)	E1-ATP (AMPPNP) (EMDB-17040) (PDB-8OP4)	E1P-ADP$^{membranous-feature}$ ($ADP-AlF_4^-$) (EMDB-17041) (PDB-8OP5)	E1P$^{cytosolic-feature}$ (AlF_4^-) (EMDB-17042) (PDB-8OP6)	E2P^{cargo} (BeF_3^-) (EMDB-17043) (PDB-8OP7)	E2.P$_i^{cargo}$ (AlF_4^-) (EMDB-17044) (PDB-8OP8)
**Data collection and processing**						
Magnification	105,000	105,000	105,000	105,000	105,000	105,000
Voltage (kV)	300	300	300	300	300	300
Total exposure (e–/Å^2)	50	40	50	50.2	50	50.2
Defocus range (μm)	-1.0 to -2.5	-1.2 to -2.6	-0.7 to -2.7	-0.5 to -2.75	-1.0 to -2.5	-0.5 to -2.75
Pixel size (Å)	1.1	0.832	0.846	0.8464	1.1	0.8464
Symmetry imposed	C1	C1	C1	C1	C1	C1
Initial particle (no.)	648,009	182,187	449,894	495,256	763,922	495,256
Final particle (no.)	196,512	115,476	151,338	107,529	249,499	185,340
Map resolution (Å)	3.5	3.2	3.4	3.7	3.5	3.4
FSC threshold	0.143	0.143	0.143	0.143	0.143	0.143
**Refinement**						
Model resolution (Å)	3.6	3.3	3.6	3.9	3.7	3.6
FSC threshold	0.5	0.5	0.5	0.5	0.5	0.5
Map sharpening *B* factor (Å^2)	-175.3	-80	-175.3	-130	-120	-130
Model composition						
Non-hydrogen atoms	8,888	8,879	9,007	8,971	9,166	9269
Protein residues	1,127	1,123	1140	1,138	1,162	1186
Ligands		MG: 1; ANP: 1	MG: 2; ALF: 1; ADP: 1	MG: 1; ALF: 1	MG: 1; BEF: 1	MG: 1; ALF: 1; CA: 1
B factors (Å^2)						
Protein	62.35	85.23	92.76	89.54	113.16	131.06
Ligand		71.04	76.44	66.24	85.13	103.81
R.m.s. deviations						
Bond lengths (Å)	0.002	0.003	0.003	0.002	0.003	0.003
Bond angles (°)	0.527	0.726	0.578	0.572	0.605	0.532
Validation						
MolProbity score	1.2	1.36	1.05	1.44	1.44	1.38
Clashscore	2.78	5.02	2.59	4.86	4.27	3.53
Poor rotamers (%)	0	0.21	0.1	0	0.20	0
Ramachandran plot						
Favored (%)	97.3	97.57	97.96	96.89	96.45	96.43
Allowed (%)	2.70	2.43	2.04	3.11	3.55	3.57
Disallowed (%)	0.00	0.00	0.00	0.00	0.00	0.00

Supplementary Table 2. Cryo-EM data collection, refinement and validation statistics

Supplementary Table 3

	UniProt	Present in	Protein name	Nr of peptide hits	Unique peptides	Cellular location	Helix type/number of helices	Location of N-terminus	Location of C-terminus	Location of majority of protein
1 TM proteins	P16547	More in SpfBef	Mitochondrial outer membrane protein OM45	40	40	Mitochondrial outer membrane	N-terminal helix	Mitochondrial intermembrane space	Cytoplasm	Cytoplasm
	P27614	More in SpfBef	Carboxypeptidase S	28	28	Vacuole	N-terminal helix	Cytoplasm	Lumen	Lumen
	Q08179	SpfBef only	Mitochondrial distribution and morphology protein 38	15	15	Mitochondrial inner membrane	Helix at residues 139-159	Mitochonrial intermembrane space	Mitochondrial matrix	Mitochondrial matrix
	P40557	SpfBef only	ER-retained PMA1-suppressing protein 1	13	13	ER	C-terminal helix	Lumen	Cytoplasm	Lumen
	P10614	SpfBef only	Lanosterol 14-alpha demethylase CYP51	7	7	ER	N-terminal helix	ER lumen	Cytoplasm	Cytoplasm
	P39006	SpfBef only	Phosphatidylserine decarboxylase proenzyme 1	7	7	ER & Mitochondrial inner membrane	Helix at residues 80-98	Mitochondrial matrix	Mitochondrial intermembrane space	Mitochondrial intermembrane space
	P46982	SpfBef only	Alpha-1,2-mannosyltransferase MNN5	7	7	Golgi	N-terminal signal peptide helix	Cytoplasm?	Lumen?	Lumen?
	P29704	SpfBef only	Squalene synthase	5	5	ER & Microsome	C-terminal TA-helix	Cytoplasm	Lumen	Cytoplasm
	P38069	SpfBef only	Alpha-1,2-mannosyltransferase MNN2	5	5	Golgi	N-terminal helix (type II)	Cytoplasm	Lumen	Lumen
	P38264	SpfBef only	SRP-independent targeting protein 3	5	5	ER & Mitochondria	N-terminal helix	Lumen	Cytoplasm	Cytoplasm
	P37299	SpfBef only	Cytochrome b-c1 complex subunit 10	4	4	Mitochondrial inner membrane	Helix at residues 22-45 out of 77	Mitochondrial matrix	Mitochondrial intermembrane space	Equal
	P38212	SpfBef only	Protein RCR1	4	4	ER	N-terminal helix	Lumen	Cytoplasm	Cytoplasm
	P38736	SpfBef only	Golgi SNAP receptor complex member 1	4	4	Golgi	C-terminal TA-helix	Cytoplasm	Lumen	Cytoplasm
	P47124	SpfBef only	Putative glycosyltransferase HOC1	4	4	Golgi	N-terminal helix (type II)	Cytoplasm	Lumen	Lumen
	P31755	SpfBef only	Initiation-specific alpha-1,6-mannosyltransferase	3	3	ER & Golgi	N-terminal helix (type II)	Cytoplasm	Lumen	Lumen
	P38288	SpfBef only	Protein TOS1	3	3	Secreted	N-terminal signal peptide helix	Cytoplasm	Extracellular space	Extracellular space

Multiple TM proteins	P25297	More in SpfBef	Inorganic phosphate transporter PHO84	24	24	Membrane	12	Cytoplasm	Cytoplasm
	Q03648	More in SpfBef	Uncharacterized protein YMR209C	24	24	Membrane	2	Unknown	Unknown
	P40098	More in SpfBef	Uncharacterized mitochondrial membrane protein FMP10	16	16	Mitochondria	2	Unknown	Unknown
	P32784	SpfBef only	Glycerol-3-phosphate O-acyltransferase 1	10	10	ER	5	Lumen	Cytoplasm
	P38325	More in SpfBef	Mitochondrial outer membrane protein OM14	8	8	Mitochondrial outer membrane	2	Unknown	Unknown
	P39952	SpfBef only	Mitochondrial inner membrane protein OXA1	8	8	Mitochondrial inner membrane	5	Mitochondrial intermembrane space	Mitochondrial matrix
	Q06169	SpfBef only	Peroxisomal membrane protein PEX30	8	8	Peroxisome	4	Unknown	Unknown
	P32476	SpfBef only	Squalene monooxygenase	7	7	ER & Microsome	2	Cytoplasm	Cytoplasm
	Q12029	SpfBef only	Probable mitochondrial transport protein FSF1	7	7	Mitochondria	4	Unknown	Unknown
	P33310	SpfBef only	ATP-dependent permease MDL1	7	6	Mitochondrial inner membrane	5	Unknown	Unknown
	P52867	SpfBef only	Dolichyl-phosphate-mannose--protein mannosyltransferase 5	6	6	ER	10	Lumen	Lumen
	Q99297	SpfBef only	Mitochondrial 2-oxodicarboxylate carrier 2	6	6	Mitochondrial inner membrane	6	Unknown	Unknown
	P47190	SpfBef only	Dolichyl-phosphate-mannose--protein mannosyltransferase 3	6	4	ER	9	Cytoplasm	Lumen
	P32564	SpfBef only	Protein SCM4	5	5	Membrane	4	Unknown	Unknown
	P47818	SpfBef only	Protein CCC1	5	5	Golgi & Vacuole	5	Cytoplasm	Lumen
	P53337	SpfBef only	ER-derived vesicles protein ERV29	5	5	ER	4	Cytoplasm	Cytoplasm
	Q03713	SpfBef only	Respiratory supercomplex factor 1	5	5	Mitochondria	5	Mitochondrial intermembrane space	Mitochondrial matrix
	P31382	SpfBef only	Dolichyl-phosphate-mannose--protein mannosyltransferase 2	5	3	ER	9	Cytoplasm	Lumen

	P36051	SpfBef only	GPI ethanolamine phosphate transferase 1	4	4	ER, Golgi & Vacuole	15	Cytoplasm	Lumen
	Q12144	SpfBef only	Pore and endoplasmic reticulum protein of 33 kDa	4	4	ER & Nucleus	6	Cytoplasm	Cytoplasm
	P19145	SpfBef only	General amino-acid permease GAP1	3	3	Cell membrane & ER	12	Cytoplasm	Cytoplasm
	P25560	SpfBef only	Protein RER1	3	3	Golgi	2	Cytoplasm	Cytoplasm

Supplementary Table 3. List of proteins that were either identified exclusively in CtSpf1/BeF3-, or among the 20 most overrepresented in CtSpf1/BeF_3^- relative to the GFP/BeF_3^- control, in the MS analysis. The table has been divided into proteins with 1 vs multiple TM helices and sorted by number of peptide hits.

Supplementary References

1 Bozzi, A. T. *et al.* Structures in multiple conformations reveal distinct transition metal and proton pathways in an Nramp transporter. *Elife* **8**, doi:10.7554/eLife.41124 (2019).

2 Wiuf, A. *et al.* The two-domain elevator-type mechanism of zinc-transporting ZIP proteins. *Sci Adv* **8**, eabn4331, doi:10.1126/sciadv.abn4331 (2022).

3 Salustros, N. *et al.* Structural basis of ion uptake in copper-transporting P(1B)-type ATPases. *Nat Commun* **13**, 5121, doi:10.1038/s41467-022-32751-w (2022).
