# Supplementary material for: The structure and function of P5A-ATPases
Source: Nat Commun. 2024 Nov 6;15:9605. doi: 10.1038/s41467-024-53757-6 (PMC11541931; doi:10.1038/s41467-024-53757-6)
Supplement: Supplementary file 2 — Reporting Summary [file 41467_2024_53757_MOESM2_ESM.pdf]

nature portfolio

Corresponding author(s): Ping Li & Pontus Goudon

Last updated by author(s): Sep 27, 2024

Reporting Summary

Nature Portfolio wishes to improve the reproducibility of the work that we publish. This form provides structure for consistency and transparency in reporting. For further information on Nature Portfolio policies, see our Editorial Policies and the Editorial Policy Checklist.

Statistics

For all statistical analyses, confirm that the following items are present in the figure legend, table legend, main text, or Methods section.

n/a	Confirmed	
☐	☒	The exact sample size (n) for each experimental group/condition, given as a discrete number and unit of measurement
☐	☒	A statement on whether measurements were taken from distinct samples or whether the same sample was measured repeatedly
☒	☐	The statistical test(s) used AND whether they are one- or two-sided<br>*Only common tests should be described solely by name; describe more complex techniques in the Methods section.*
☒	☐	A description of all covariates tested
☒	☐	A description of any assumptions or corrections, such as tests of normality and adjustment for multiple comparisons
☒	☐	A full description of the statistical parameters including central tendency (e.g. means) or other basic estimates (e.g. regression coefficient) AND variation (e.g. standard deviation) or associated estimates of uncertainty (e.g. confidence intervals)
☒	☐	For null hypothesis testing, the test statistic (e.g. F, t, r) with confidence intervals, effect sizes, degrees of freedom and P value noted<br>*Give P values as exact values whenever suitable.*
☒	☐	For Bayesian analysis, information on the choice of priors and Markov chain Monte Carlo settings
☒	☐	For hierarchical and complex designs, identification of the appropriate level for tests and full reporting of outcomes
☒	☐	Estimates of effect sizes (e.g. Cohen's d, Pearson's r), indicating how they were calculated

Our web collection on statistics for biologists contains articles on many of the points above.

Software and code

Policy information about availability of computer code

Data collection	Cryo-EM datasets were collected on Titan Krios electron microscopes with a Falcon3 detector or a Gatan K3 detector.<br>The LC-MS detection was performed on Tribrid mass spectrometer Fusion equipped with a Nanospray Flex ion source and coupled with an EASY-nLC 1000 ultrahigh pressure liquid chromatography (UHPLC) pump (Thermo Fischer Scientific).
Data analysis	Cryo-EM data for E1-ATP state was processed with cryosparc v3.0.1 and Cryo-EM data for other states were processed with cryosparc v3.3.2. Model building and refinement were performed using USCF chimera 1.14, Wincoot 0.9.2 and phenix 1.20.1. Figures were generated using USCF chimera v1.14, ChimeraX v1.5 and pymol v2.3.4. Sequence alignments were performed using Cluster Omega (online), and visualized using ESPript 3.0. The Mass spectrum data analysis was performed using Maxquant 2.0.2 and post-processing was performed in Perseus 1.6.15.

For manuscripts utilizing custom algorithms or software that are central to the research but not yet described in published literature, software must be made available to editors and reviewers. We strongly encourage code deposition in a community repository (e.g. GitHub). See the Nature Portfolio guidelines for submitting code & software for further information.

Data

Policy information about availability of data

All manuscripts must include a data availability statement. This statement should provide the following information, where applicable:

- Accession codes, unique identifiers, or web links for publicly available datasets
- A description of any restrictions on data availability
- For clinical datasets or third party data, please ensure that the statement adheres to our policy

The sequence of P5A-ATPase CtSpf1 is available in the following link:
https://www.uniprot.org/uniprotkb/G0S4Z4/entry
Cryo-EM maps have been deposited in the Electron Microscopy Data Bank (EMDB) under accession codes:
EMD-17039 (E1), EMD-17040 (E1-ATP), EMD-17041 (E1P-ADPmembranous-feature), EMD-17042 (E1Pcytosolic-feature), EMD-17043 (E2Pcargo) and EMD-17044 (E2.Picargo).
The atomic coordinates have been deposited in the Protein Data Bank (PDB) under accession codes 8OP3 (E1), 8OP4 (E1-ATP), 8OP5 (E1P-ADPmembranous-feature), 8OP6 (E1Pcytosolic-feature), 8OP7 (E2Pcargo) and 8OP8 (E2.Picargo).
The mass spectrometry proteomics data have been deposited to the ProteomeXchange Consortium via the PRIDE partner repository with the dataset identifier PXD042401.

Research involving human participants, their data, or biological material

Policy information about studies with human participants or human data. See also policy information about sex, gender (identity/presentation), and sexual orientation and race, ethnicity and racism.

Reporting on sex and gender	n/a
Reporting on race, ethnicity, or other socially relevant groupings	n/a
Population characteristics	n/a
Recruitment	n/a
Ethics oversight	n/a

Note that full information on the approval of the study protocol must also be provided in the manuscript.

Field-specific reporting

Please select the one below that is the best fit for your research. If you are not sure, read the appropriate sections before making your selection.

☒ Life sciences ☐ Behavioural & social sciences ☐ Ecological, evolutionary & environmental sciences

For a reference copy of the document with all sections, see nature.com/documents/nr-reporting-summary-flat.pdf

Life sciences study design

All studies must disclose on these points even when the disclosure is negative.

Sample size	No specific method was applied to determine the sample size. The complete cryo-EM datasets were collected by microscopy with available time and each collected dataset was sufficient to obtain high resolution maps. Three independent biological samples for different states were prepared for the mass spectrum study.
Data exclusions	In the cryo-EM data analysis, bad micrographs were excluded with low CTF fitting resolution and bad particles were excluded to generate the high resolution maps.
Replication	Multiple grids were prepared for each sample state and data was collected with selected grid for individual state. Samples for the mass spectrum study were purified with 3 independent biological replicates. Functional assay experiments in this study have been repeated and findings are reproducible.
Randomization	No randomization applied in this study. For the cryo-EM data analysis, particles were processed automatically by Cryosparc. The samples were prepared with different states for the mass spectrum study and no randomization was assigned.
Blinding	No blinding was applied in this study as no group allocation was used and blinding is not relevant to the cryo-EM analysis and mass spectrum data analysis.

Reporting for specific materials, systems and methods

We require information from authors about some types of materials, experimental systems and methods used in many studies. Here, indicate whether each material, system or method listed is relevant to your study. If you are not sure if a list item applies to your research, read the appropriate section before selecting a response.

Materials & experimental systems

n/a	Involved in the study
☒	☐ Antibodies
☐	☒ Eukaryotic cell lines
☒	☐ Palaeontology and archaeology
☒	☐ Animals and other organisms
☒	☐ Clinical data
☒	☐ Dual use research of concern
☒	☐ Plants

Methods

n/a	Involved in the study
☒	☐ ChIP-seq
☒	☐ Flow cytometry
☒	☐ MRI-based neuroimaging

Eukaryotic cell lines

Policy information about cell lines and Sex and Gender in Research

Cell line source(s)	S. cerevisiae (PAP1500) strain was used for the protein production and S.cerevisiae (BY4741) strain was used for functional assay.
Authentication	Not authenticated
Mycoplasma contamination	no
Commonly misidentified lines (See ICLAC register)	n/a
